# Efficacy of Guizhi Fuling Wan for primary dysmenorrhea: protocol for a randomized controlled trial

**DOI:** 10.1186/s13063-021-05834-0

**Published:** 2021-12-18

**Authors:** Yun Du, Yatong Li, Xianyun Fu, Chenjie Li, Luo Yanan

**Affiliations:** 1 The Second people’s Hospital of Yichang, China Three Gorges University, Yichang, Hubei China; 2 The Department of Traditional Chinese Medicine, Medical College of China, Three Gorges University, Yichang, 443003 Hubei China

**Keywords:** Guizhi Fuling Wan, Primary dysmenorrhea, Randomized controlled trial, Placebo, Control

## Abstract

**Background:**

Primary dysmenorrhea (PD) is one of the main gynecological complaints in women of child-bearing age, but limited effective treatments are available. Guizhi Fuling Wan (GFW), one of the most widely known traditional Chinese medicine (TCM) formulations, has been commonly used in clinical practice to treat gynecological disorders in China. In recent years, a growing number of studies have shown that GFW is beneficial for patients with PD. However, the quality of evidence is limited, and there are few studies on specific TCM syndromes of GFW for PD. Therefore, we plan to conduct a randomized controlled trial to explore the efficacy and safety of GFW for PD patients with heat-burning blood-stasis syndrome.

**Methods and analysis:**

The clinical study is a randomized, double-blinded, placebo-controlled trial. Eligible patients will be randomly assigned to the GFW group (treated with GFW) and the control group (treated with a matching placebo) in a 1:1 ratio for three menstrual cycles with a 3-month follow-up. The primary outcome will be the mean change of pain intensity measured by the visual analog scale (VAS). The secondary outcomes will include the Cox Menstrual Symptom Scale (CMSS), the Self-rating Depression Scale (SDS), the Self-rating Anxiety Scale (SAS), and the TCM syndrome scale. Adverse events will also be reported.

**Discussion:**

This randomized trial will be the first rigorous study designed to assess the efficacy and safety of GFW in treating PD with heat-burning blood-stasis syndrome. The finding of this study will provide an objective clinical basis for the use of GFW for PD in the future.

**Trial registration:**

Chinese Clinical Trial Registry ChiCTR2000034118. Registered on 24 June 2020

**Supplementary Information:**

The online version contains supplementary material available at 10.1186/s13063-021-05834-0.

## Background

Primary dysmenorrhea (PD) is a common gynecological disorder among reproductive women. It is estimated that 40 to 75% of women may suffer from cramping pain in the lower abdomen, even with associated symptoms such as weakness, diarrhea, nausea, and headache [1–4]. Approximately 75% of reproductive women are affected by PD at some point in their lives. Meanwhile, absenteeism from school and work due to severe PD has been as high as 14%, seriously affecting their quality of life [4, 5]. Currently, the most commonly used clinical pharmacological therapies are non-steroidal anti-inflammatory drugs (NSAIDs) and oral contraceptives [6, 7]. Given that 10–20% of PD patients do not respond to the treatment of NSAIDs and that some patients cannot tolerate their side effects or are unsuitable for their indications [8, 9], alternative therapies have become an important addition for PD treatment, in which Chinese herbal medicine (CHM) has played the essential role. CHM, as an essential part of traditional Chinese medicine (TCM), has been widely used in the clinical treatment of PD, especially in China. GFW is one of the most widely known CHM formulas for the treatment of PD and consists of five herbs, including Cinnamon Twig, Poria cocos, Cortex Moutan, Radix Paeoniae Rubra, and Peach kernel. Originating from ancient simple philosophy and empirical medicine, the effect of GFW has been proven in clinical practice and widely used to treat PD, as well as other gynecological diseases such as endometriosis, uterine fibroids, pelvic inflammation, and mastocytosis [10]. In recent basic researches, GFW’s pharmaceutical ingredient had been shown to suppress uterus contraction, prostaglandin level reduction, and correction of luteal insufficiency, demonstrating the potential of promising drugs for PD [11–13]. It also suggested that the GFW had therapeutic effects on PD rats via the regulation of multiple metabolic pathways [14]. Several clinical trials have also shown promising results and no severe adverse reactions of GFW in the treatment of PD [15–18]. However, the relevant studies were prospective long-term clinical observational studies, while RCTs were rare [19]. A few earlier RCTs followed the 2010 version of the CONSORT standard. They rarely used the latest CONSORT extension for Chinese herbal medicine formulas 2017, which can better ensure the quality and authenticity of TCM researches [20]. In addition, most trials did not diagnose patients with TCM dialectical classification, which is the core of TCM treatment practice [21]. A systematic review suggested that GFW was effective in treating PD. However, the authors concluded that the clinical results in these studies were tentative and should be interpreted with caution due to the lack of quality of the included studies [22]. Therefore, to evaluate the efficacy and safety of GFW for PD with the heat-burning blood-stasis syndrome in a small sample size and to reduce the subjective expectations of the study subjects and investigators, we aim to design a rigorous randomized, double-blinded, placebo-controlled trial.

## Methods

### Design and setting

This study is a randomized, double-blind, and placebo-controlled exploratory trial with two parallel groups. The study is designed using Consolidated Standards of Reporting Trials guidelines Extension for Chinese Herbal Medicine Formulas 2017 (CONSORT CHM Formula) [20] and adhere to the Declaration of Helsinki (2013 version). Meanwhile, we designed the study according to the Standardized Protocol Items: Recommendations for Interventional Trials (SPIRIT) statement [23] (details see Additional file 1). The study protocol has been approved by the Ethical Committee of The Second People’s Hospital of Yichang (No. 2020001) and has been registered at the Chinese Clinical Trials Registry in 2020 (No. ChiCTR2000034118.) (details see https://trialsearch.who.int/Trial2.aspx?TrialID=ChiCTR2000034118 and Additional file 2).

**Fig. 1:**
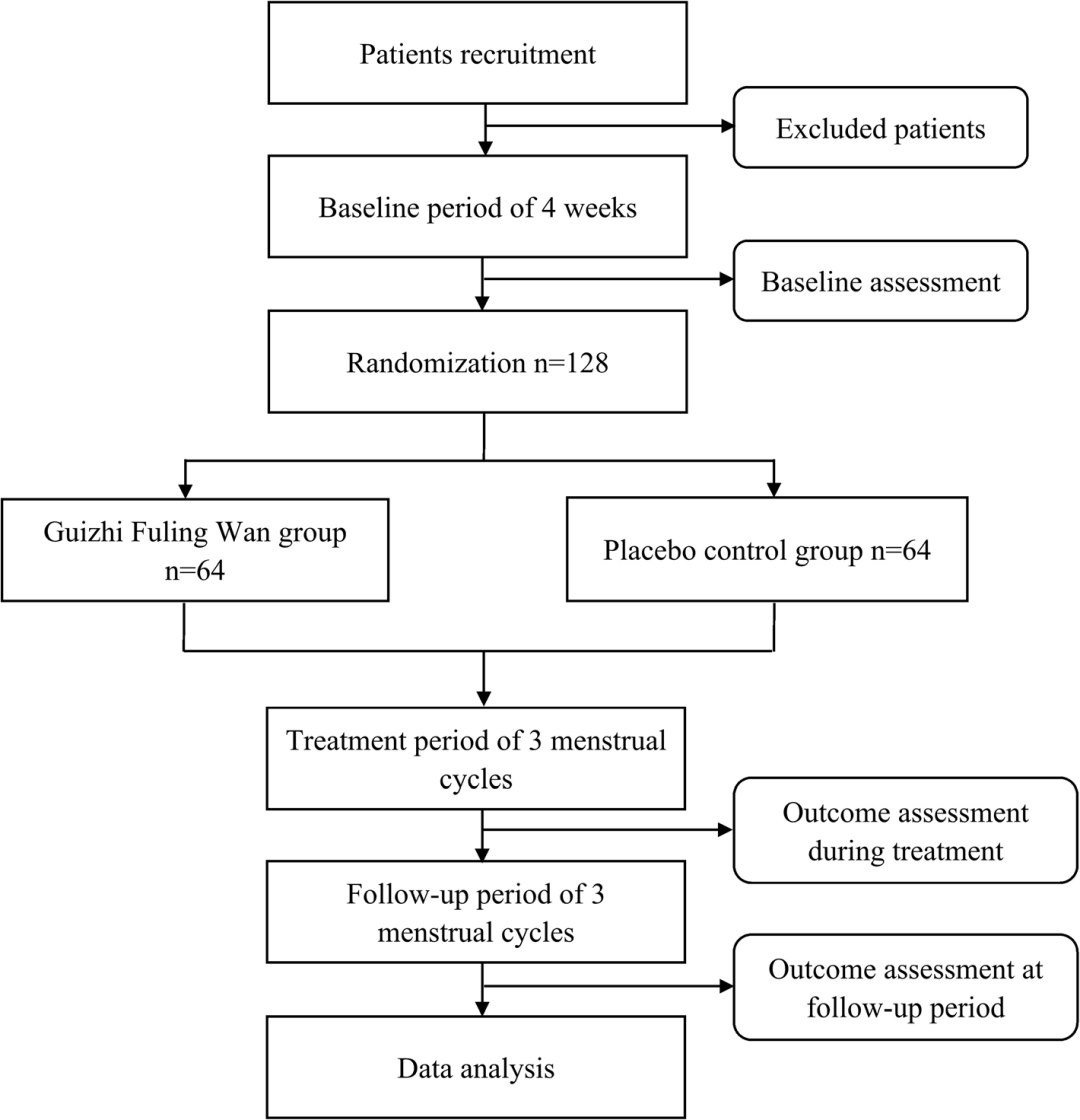
Flowchart of the trial

**Fig. 2:**
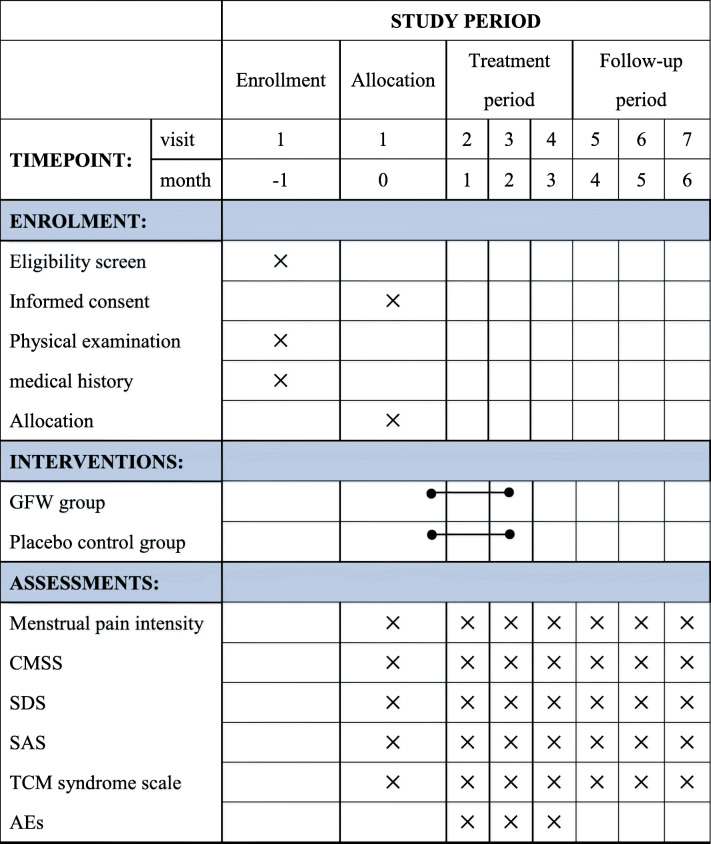
Schedule of enrolment, interventions, assessments, and data collection. *CMSS* Cox Menstrual Scale, *SDS* the Self-rating Depression Scale, *SAS* the Self-rating Anxiety Scale, *SF-12* the 12-item Short-Form Health Survey, *AEs* adverse events

The trial is being conducted at the Second People’s Hospital of Yichang. Participants meeting the inclusion criteria will be randomly allocated to either the GFW group (treated with GFW) or the control group (treated with a matching placebo) in a 1:1 ratio. The total observation period will be 7 months, including a 1-month baseline period, a 3-month treatment period, and a 3-month follow-up period. The intensity of menstrual pain and the acute medication use will be asked to record in dysmenorrhea diaries throughout the trial. Assessments will be conducted at baseline and at 1, 2, 3, 4, 5, and 6 months postmenstrual cycles. The flow chart is shown in Figs. 1 and 2 illustrates the schedule of enrolment, interventions, assessments, and data of participants.

### Participants

Potential participants who meet the inclusion criteria but do not meet any exclusion criteria as diagnosed by two TCM gynecologists will be asked to meet face-to-face with a research assistant to discuss the study. Eligible patients interested in participating will undergo a series of condition assessments and safety evaluations. After giving informed consent, they will be randomized into two groups.

### Inclusion criteria

Participants meeting the following criteria will be included: (1) nulliparous women aged 16 to 30 years old; (2) meeting the diagnostic criteria for PD in Chinese Obstetrics And Gynecology (3rd edition); (3) meeting the TCM diagnosis of “heat-burning blood-stasis syndrome”; (4) pain symptoms persisted for 6 months to 15 years; (5) VAS score of menstrual pain ≥ 40 mm for at least three consecutive menstrual cycles; (6) patients with the syndrome of heat burning and blood stasis; and (7) signed informed consent form.

### Exclusion criteria

Participants will be excluded if they meet any of the following criteria: (1) unable to complete or comply with the study; (2) imaging examination suspects secondary dysmenorrhea; (3) pregnancy during the trial; (4) inability to cooperate with the questionnaires due to intellectual or mental disorder; (5) history or presence of clinically relevant severe cardiovascular diseases, liver, kidney dysfunction, or psychiatric disease; (6) treatment of PD with analgesics, antidepressants, or psychotropic drugs in the past month; (7) irregular menstrual cycle (beyond the typical range of 21–35 days); and (8) are participating in the other clinical trials of drugs.

### Recruitment and informed consent

PD patients will be recruited through the gynecology clinic of the Second People’s Hospital of Yichang, media releases from Three Gorges University, and online promotion. A 1-month baseline assessment will be needed before randomization, and two research assistants will visit the gynecology department of the hospital twice a week to identify and invite eligible patients to participate in the study. During the baseline assessment period, subjects who meet the acceptance criteria and agree to join the study will be explained the general study process and the responsibilities of the participants and researchers. In addition, patients will be informed of all details of the study and then sign a written informed consent form, which will also be signed and witnessed by the research assistants. No additional biological samples will be obtained to be stored for use in this study. Sample patient informed consent is seen in Additional file 3.

### Randomization and allocation concealment

Eligible participants will be randomly assigned to either the treatment group or the control group by the simple randomization in a 1:1 ratio. Random numbers are computer-generated using the SPSS Statistics for Windows, version 22.0 (SPSS Inc., Chicago, III., USA) by an independent researcher, who will not be involved in patient care, outcome assessment, data collection, or data analysis. For allocation concealment, the practice is to enclose assignments in sequentially numbered, sealed envelopes. The sealed envelopes cannot be opened unless a new participant is included in the trial and will be kept in a safe place in numerical order until the study is completed. Complete separation from the individuals involved in the steps before enrolment from those involved in the allocation concealment. All patients who agree to participate and meet the inclusion criteria will be randomized. Randomization will be requested by the staff member responsible for recruitment. In return, the clinical center will give a closed opaque envelope with randomization numbers inside. The therapist will open the envelope and will find the treatment to be administered to that patient. The staff member responsible for recruitment will not be allowed to receive information about the group assignment.

### Blinding

The participants, clinical researchers, outcome evaluators, data manager, and statisticians will be blinded to the treatment allocations during the study. The placebo granule is matched to the GFW granule in color, taste, odor, and outer packaging to ensure that the researchers and patients involved in the study are entirely unaware of the identity of the treatment being given. Blinding will not be disclosed until the end of the study unless a serious adverse event (SAE) occurs and their specific treatment needs to be known to provide emergency assistance. Details of the unblinding, such as the reason and date, will be recorded on a case report form (CRF).

### Interventions

Participants in the GFW group will receive GFW (herb granule, 30 g per packet) treatment. It has the effect of clearing heat and dispelling dampness, blood circulation, removing blood stasis, and clearing away heat and blood. It consists of five natural herbs: Guizhi (Cinnamon Twig) 6 g, Fuling (Poria Cocos) 6 g, Mudanpi (Cortex Moutan) 6 g, Chishao (Radix Paeoniae Rubra) 6 g, and Taoren (Peach Kernel) 6 g [24]. Participants in the control group will receive a GFW-matched placebo consisting of Maiya (Hordei Fructus Germinatus) without analgesic effects. The dosage form, color, appearance, and packaging of the GFW-matched placebo are consistent with those of GFW in the treatment group [25]. Both granules are provided and quality controlled by Beijing Tcmages Pharmaceutical Co. Ltd. (Beijing, China) and distributed by the Second People’s Hospital of Yichang. All drugs are packaged and labeled uniformly.

According to clinical experience, a total of 3 menstrual cycles were treated, each cycle from 7 days before each menstruation to the 3rd day of menstruation, once in 12 h, 30 g each time for ten days. All the participants will be advised not to take NSAID drugs or COCs for PD during the baseline, treatment, and follow-up periods. However, physical therapies (e.g., hot compress and moxibustion) are allowed if the pain is intolerable and the outcome assessment is not scheduled in the next 48 h [26].

To improve patient compliance with the intervention protocol, patients will be required to record all these treatments and keep the package after each dose. The formula calculates the compliance rate: Compliance rate = (actual dosage/expected dosage) × 100%. Patients with a compliance rate ≥ 80% are considered to have good compliance. In addition, face-to-face compliance reminders will be held at each medication distribution to emphasize dosing instructions, the purpose of treatment, etc., especially for those participants who do not achieve 80% of the compliance rate.

Participants are free to withdraw from the trial if they feel they are not getting better. Also, if they develop a serious illness or a serious adverse event related to the study, allocated interventions will discontinue. Participants can continue to receive health counseling services from clinicians after completing the study. Doctors should offer alternative treatments for patients who have completed the trial without remission, such as combined conservative treatments like acupuncture.

### Outcome measurements

The time points for all outcomes are at baseline and at 1, 2, 3, 4, 5, and 6 months postmenstrual cycles.

#### Primary outcome

The primary outcome is the mean menstrual pain intensity change measured by visual analog scale (VAS) from baseline to 6 months. The VAS is a valid and reliable tool that has been widely used in various clinical trials involving patients with PD [27]. Participants will be asked to indicate a perception of pain intensity scored from 0 to 100 (0, no pain; 100, maximum).

#### Secondary outcomes

The secondary outcomes are (1) the change of the mean Cox Menstrual Symptom Scale (CMSS) score from baseline to 6 months, (2) the change of the mean Self-rating Depression Scale (SDS) [28] score from baseline to 6 months, (3) the change of the mean Self-rating Anxiety Scale (SAS) [29] score from baseline to 6 months, and (4) the change in the proportion of the TCM syndrome scale score from baseline to 6 months. The CMSS is a compact questionnaire for the evaluation of menstrual pain symptoms [30]. It consists of 18 items, each of which is divided into five levels according to the severity of symptoms (a score of 0 indicates no discomfort; 1 for mild discomfort; 2 for moderate discomfort; 3 for severe discomfort; and 4 for extremely severe), including an evaluation of the general frequency and average severity of menstrual symptoms [31]. The SAS and SDS are scoring tools for depression and anxiety disorders, respectively, and are widely used in clinical PD patients because of the emotional changes that often accompany PD. The SAS questionnaire for self-rated includes 20 items, and each is answered on a 4-point scale ranging from 0 (no time) to 4 (most of the time), with a total score of more than 40 indicating a clinically relevant anxiety disorder [32]. The higher the score, the more severe the anxiety. The SDS is a self-rated scale for evaluating the severity of depression. The statistical indicators of SAS include 20 questions with a total score ranging from 20 to 80. A score above 40 indicates depressive symptoms, and the higher the score, the more pronounced the tendency to depression [33]. The TCM syndrome scale is a questionnaire for the evaluation of TCM symptoms. It includes 9 items with a total score ranging from 0 to 26, and the higher the score, the more serious the degree of TCM syndrome [34]. The assessments will be performed in a separate space in the outpatient department. All of the outcome assessors will be trained in conducting interviews and performing measurements before the study.

### Assessment of safety

All unexpected or unfavorable events affecting patients in the study, such as heavy menstrual flow, nausea, bloating, diarrhea, and stomach upset, associated with treatment [18, 19], will be recorded in detail in the CRFs and followed up for the duration of the study. Investigators will confirm the relatedness of adverse events and treatment and report treatment-related adverse events. In case of adverse reactions, the clinical observer can decide whether to stop the trial depending on the patient’s condition. In the event of any serious adverse events (SAEs), emergency medical assistance will be sought. The outcome will be assessed at 1, 2, and 3 months after randomization.

### Sample size

The sample size estimation was based on the mean difference in the change of the menstrual pain intensity from baseline. Based on previous studies and our pilot trial, we made the following assumptions in calculating the sample size: a mean difference of 1 between two groups, SD of total score of 1.67, a ratio of 1:1 between the two groups, a two-sided significance level of 0.025 and a power of 0.9 [35, 36]. With these assumptions, a minimum sample size of 116 patients (58 patients in each group) is needed. Considering an estimated 10% dropout rate, the estimated sample size was 128 patients (64 patients per group).

### Data collection and management

This trial mainly involved randomized group data, baseline data, post-intervention outcome data, follow-up data, and statistical analysis result data. Randomized group data are provided and managed by the investigator responsible for the random grouping of the trial. Baseline data, post-intervention outcome data, and follow-up data are collected by dedicated data collection researchers. Statistical analysis result data are collected and provided by the researcher in charge of data statistical analysis. In order to maximize the completeness of data collection, we will enhance communication with patients. We will strengthen our contact with patients by phone or social media to remind and monitor their medications, in addition to the face-to-face reminders at each visit. Free examinations and treatments are offered during the study, and visit times are flexible according to the patient’s schedule as far as possible. Furthermore, we will hold regular TCM health lectures at our clinical trial center to engage participants. All participants are assessed at baseline and 1, 2, 3, 4, 5, and 6 months after randomization. Work training will be held before the start of the project to provide detailed training and explanation of the trial protocol, standard operating procedures (SOPs) for study operations, and the content of case report forms (CRFs). Well-trained assessors, blinded to the treatment assignment, will use CRFs to collect clinical data at each visit. The person in charge of CRFs checks the daily completion and files the case report form in summary after standardization. Supervisors also should randomly check the CRFs at any time. Data will be recorded on the paper version of CRFs by designated outcome assessors and double entered in the electronic CRFs. Any incomplete data will be recorded as unknown, missing, or not applicable. All study-related information, such as participants’ medical records, will be kept securely in locked file cabinets with limited access. All private subject data are encrypted and protected, visible only to the project’s principal investigators, and used only for the research of the project. Principal investigators will be granted access to the cleaned dataset. To ensure confidentiality, data dispersed to project team members will be kept confidential to any identifiable participant information.

### Biological specimens

Not applicable in this study.

### Data analysis

At the end of all measurements’ data collection, a skilled statistician blinded to group allocation will be performed by SPSS Statistics for Windows, version 22.0 (SPSSInc., Chicago, III., USA). We will descriptively summarize patient characteristics, outcome variables, and adverse events. Continuous variables, such as age and disease course, will be presented as mean ± standard deviation (*SD*) and analyzed by an independent-sample *T*-test. Quantitative variables such as baseline variables of socio-demographic data will be analyzed using *χ*^2^ or Mann-Whitney *U* tests. The repeated measurement variables, such as the primary and second outcomes, are analyzed by repeated-measures analysis of variance (ANOVA) or generalized estimation equations (GEE).

Both the full analysis set (FAS) and the per-protocol analysis set (PPS) will be used simultaneously to evaluate the effect and safety of GFW for PD. The FAS is for all participants who complete randomization and are assigned to receive treatment. The PPS is for the individuals who receive the assigned treatment according to the protocol and complete the entire study. For missing data, we will use the last-observation-carried-forward method. Although no interim analysis of outcomes is planned, a summary of the enrolment progress, AEs, and protocol deviations will be provided to the data monitoring committee (DMC) members. The trial should be terminated if there is clear evidence that SAEs are associated with the intervention or develop a serious illness. Statistical significance will be set at *P* < 0.05.

### Oversight and monitoring

We have developed the following committees for the trial. Study principal investigator: Chenjie Li. Steering committee: Chenjie Li (chair), Yanan Luo, Yun Du, Yatong Li, and Xianyun Fu. Methods Center staff: Chenjie Li, Yanan Luo, Yun Du, Yatong Li, and Xianyun Fu. Data monitoring committee: Ping Mao (chair), Kun Wang, and Minmin Chen. Site audit committee: Jiaqi Cui and Wanyu Xia. In addition, an independent data monitoring committee (DMC) will be established to monitor the trial’s quality and compliance and ensure the safety of participating patients. The DMC will be composed of three experts with expertise in TCM, PD management, and trial methodology, who will examine the data every 6 months and determine if a trial should be modified or discontinued. All of them are independent of the sponsor and have no competing interests. Interim analyses will be performed by independent statisticians and reported to the DMC, which can obtain these interim results and make the final decision to terminate the trial.

### Protocol amendments

Any modification to the protocol that may affect the conduct of the study, the potential benefit to the patient, or may affect patient safety requires a formal modification to the protocol. Such change will be approved by the sponsor and the Ethical Committee of the Second People’s Hospital of Yichang before implementation.

### Quality control

The protocol has been reviewed several times by experts in TCM gynecology and methodology. Before the start of the trial, all study staff will be required to attend SOP training to ensure that each study staff member is familiar with the trial. During the trial, the study team holds workshops every 2 months to assess the trial progress, clinical feasibility, and side effects, etc. Supervisors also should regularly check the progress of the trial.

## Discussion

GFW, the classical CHM formula, is regarded as a promising therapeutic Chinese herb for PD, which reduces hyperalgesia, muscle contraction, vasoconstriction, inflammatory pain, and uterine ischemia by regulating the overproduction of prostaglandins (PGs) [11–13]. However, the scientific basis remains relatively limited due to methodological shortcomings, insufficient reports, or the lack of syndrome differentiation in previous studies [19]. Therefore, we intend to design a randomized controlled, double-blind trial to evaluate the efficacy and safety of GFW in treating PD with the heat-burning blood-stasis syndrome as a complementary and alternative therapy.

Treatment based on syndrome differentiation has always been the core of TCM clinical diagnosis and treatment. Accurate syndrome differentiation and treatment not only can guide the use of medication but also improve the efficacy. Currently, researchers are paying increasing attention to the treatment based on syndrome differentiation and gradually introducing it into clinical trials [21]. We found that GFW is effective in treating PD through long-term clinical observation, especially in patients with “heat-burning blood-stasis syndrome.” Therefore, this study includes the PD with the heat-burning blood-stasis syndrome. To ensure the reliability of TCM dialectical classification, two experienced TCM doctors will provide patients with TCM diagnoses.

Our study also has a few limitations. First, to improve patient compliance and facilitate patient recruitment, we enrolled patients from China Three Gorges University, which could lead to selective bias to some extent. However, the university is a comprehensive university with students from all over the country. Second, the placebo and GFW are identical in texture, dosage, and appearance but have subtle differences in odor and taste, which may have some implementation bias.

In sum, this trial is the first study designed to demonstrate the efficacy and safety of GFW in treating PD patients with the heat-burning blood-stasis syndrome compared with a placebo. This trial’s scientific and rigorous methodological design is expected to provide reliable evidence for the efficacy and safety of GFW in treating PD and provide a reference for clinical practice.

## Trial status

Currently, the protocol version number is version 1.0, registered on June 24, 2020. At the time of manuscript submission, the study had been actively recruiting subjects and ongoing.

## Supplementary Information

**Additional file 1.** Trial registration data.

**Additional file 2 **SPIRIT Checklist for *Trials.*

**Additional file 3.** Sample patient informed consent.

**Additional file 4.** Ethical Approval Document.

**Additional file 5.** Funding Documentation.

## Data Availability

At the end of the study, a completely de-identified data set is scheduled to be delivered to an appropriate data archive.

## Abbreviations

CMSS: Cox Menstrual Scale
SDS: Self-rating Depression Scale
SAS: Self-rating Anxiety Scale
SF-12: The 12-item Short-Form Health Survey
AEs: Adverse events
SAE: Serious adverse event
HT: Hashimoto’s thyroiditis
TPOAb: Antithyroperoxidase antibodies
TCM: Traditional Chinese medicine
PNHM: Penetration needling of Hand-Yangming meridian
SPIRIT: Standard Protocol Items: Recommendations for Interventional Trials
STRICTA: Standards for Reporting Interventions in Clinical Trials of Acupuncture
TRAb: Thyroid-stimulating hormone receptor antibody
TgAb: Antithyroglobulin antibodies
SOP: Standard operation procedure
CRFs: Case report forms
SOPs: Standard operating procedures
