# Correction to: Efficacy of Guizhi Fuling Wan for primary dysmenorrhea: protocol for a randomized controlled trial

**DOI:** 10.1186/s13063-021-05958-3

**Published:** 2021-12-29

**Authors:** Yun Du, Yatong Li, Xianyun Fu, Chenjie Li, Yanan Luo

**Affiliations:** 1 The Second people’s Hospital of Yichang, China Three Gorges University, Yichang, Hubei China; 2 The Department of Traditional Chinese Medicine, Medical College of China, Three Gorges University, Yichang, 443003 Hubei China

**Correction to: Trials 22, 933 (2021)**

**https://doi.org/10.1186/s13063-021-05834-0**

Following the publication of the original article [1], we were notified of a typo in the fifth author’s name:

- Originally published name: Yanan Lou

- Corrected name: Yanan Luo

The original article has been corrected.
