# Supplementary material for: Efficacy of Guizhi Fuling Wan for primary dysmenorrhea: protocol for a randomized controlled trial
Source: Trials. 2021 Dec 18;22:933. doi: 10.1186/s13063-021-05834-0 (PMC8684114; doi:10.1186/s13063-021-05834-0)
Supplement: Supplementary file 1 — Additional file 1. Trial registration data. [file 13063_2021_5834_MOESM1_ESM.pdf]

**Table2 | Trial registration data**	
**Data category**	**Information**
Primary registry and trial identifying number	ChiCTR2000034118
Date of registration in primary registry	25 June 2020
Secondary identifying numbers	ChiMCTR2000003429
Source(s) of monetary or material support	National Natural Science Foundation of China (No. 81973897)
Primary sponsor	The Second People's Hospital of Yichang
Secondary sponsor(s)	/
Contact for public queries	Chenjie Li
Contact for scientific queries	Chenjie Li The Second People's Hospital of Yichang, Hubei, China
Public title	Efficacy of Guizhi Fuling Wan for Primary dysmenorrhea
Scientific title	Efficacy of Guizhi Fuling Wan for Primary dysmenorrhea: protocol for a randomised controlled trial
Countries of recruitment	China
Health condition(s) or problem(s) studied	Guizhi Fuling Wan, Primary dysmenorrhea
Intervention(s)	Treatment group: GFW (60g GFW per day)
	Control group: Maiya (60g Maiya per day)
Key inclusion and exclusion criteria	Inclusion criteria: nulliparous women of 16 to 30 years old, meet the diagnostic criteria of PD by Chinese Obstetrics And Gynecology (3rd edition) . . .
	Exclusion criteria: unable to complete or comply with the study. . .
	Age minimum:16<br>Age maximum:30<br>Gender: Female
Study type	Interventional
	Allocation: randomized; Intervention model: parallel assignment; Masking: double blind . . .
	Primary purpose: verification
	Phase:0
Date of first enrolment	September 2020
Target sample size	128
Recruitment status	Recruiting
Primary outcome(s)	visual analog scale (VAS) scores
Key secondary outcomes	The TCM syndrome score, Cox Menstrual Symptom Scale (CMSS), Self-rating Depression Scale (SDS), Self-rating Anxiety Scale(SAS)
