# Supplementary material for: Efficacy of Guizhi Fuling Wan for primary dysmenorrhea: protocol for a randomized controlled trial
Source: Trials. 2021 Dec 18;22:933. doi: 10.1186/s13063-021-05834-0 (PMC8684114; doi:10.1186/s13063-021-05834-0)
Supplement: Supplementary file 3 — Additional file 3. Sample patient informed consent. [file 13063_2021_5834_MOESM3_ESM.pdf]

受试者知情同意书

亲爱的患者：

您的医生已经确诊您患有原发性痛经。我们将邀请您参加一项药物治疗原发性痛经的随机对照临床试验。

在您决定是否参加这项研究之前，请尽可能仔细阅读以下内容。它可以帮助您了解该项研究以及为何要进行该项研究，研究的程序和期限，参加研究后可能给您带来的益处、风险和不适。如仍有不清楚之处，请询问您的医生，他或她将解答您提出的疑问。如果您愿意，您也可以和您的亲属、朋友一起讨论，或者请医生给予解释，帮助您做出决定。

研究介绍

一、研究背景和研究目的

痛经的发生率在年轻患者中高达 80%，反复发作的痛经除影响患者生活质量外，也可能并发盆腔慢性炎症甚至致原发性不孕症的发生。常用改善痛经的方法包括物理治疗、西药及中药治疗。物理治疗如热敷仅能改善轻度痛经症状；西药如布洛芬虽能部分改善症状，但其副作用较大，停药后复发率高；中医药的治疗因其疗效较好、副作用小，可改善体质、显著减轻伴随症状、降低复发率而在临床广泛运用。

在中医学理论中，痛经的核心病机为“瘀血证”“不通而痛”，而瘀血的发生因为病因的不同而分为寒凝血瘀、气滞血瘀、气虚血瘀、湿热瘀结和热灼血瘀等多种证型。瘀血证是痛经发生的核心病机，“桂枝茯苓丸”（桂枝、茯苓、赤芍、丹皮、桃仁）具有药物组方简单、平和的特点，为中医经典活血化瘀方，有悠久的历史，临床运用广泛，疗效较好。

药物介绍：桂枝茯苓丸来源于中医经典《金匮要略》，在临床广泛使用，本课题组针对湿热瘀结证型、结合临床运用，采用桂枝温通经脉而行瘀滞；桃仁化瘀；茯苓消痰利水，渗湿健脾；将方中白芍易为赤芍，与丹皮共用，散血行瘀，清退瘀久所化之热，增强凉血活血之效。诸药合力缓缓活血化瘀，以达治疗痛经的疗效。本课题组临床实践表明，桂枝茯苓丸具有化瘀生新、调和气血的效果，对缓解痛经、胃肠道伴随症状症状均有一定的疗效，并对月经量无显著影响。课题组前期的基础研究也证实，活血化瘀药物可显著改善子宫内膜缺氧状态、减少氧化应激炎症，有多靶点治疗作用。

本研究的目的是：采用大样本的临床研究，使用桂枝茯苓丸对热灼血瘀证原发性痛经的临床治疗效果。

该试验将有128名受试者参加。

二、哪些人不宜参加研究

凡符合下述任何一条的患者，均不宜参加该试验：

1.半年内不能完成本研究治疗方案的患者；

2.曾经参与或正在参与其它临床试验者；

3.月经周期不规律(超出 21-35 天的典型范围)；

4.近半年有妊娠计划的妇女；

5.有精神障碍及智能障碍不能配合问卷调查者；

6.合并有严重的心血管疾病、肝肾功能损害者、免疫缺陷、糖尿病、痛风、血液病者；心理疾病或者危及生命的情况；

7.正在使用镇痛药物。

三、如果参加研究将需要做什么？

1. 在您入选研究前，您将接受以下检查以确定您是否可以参加研究：

医生将询问、记录您的病史，并进行中医证型的辩证。您需要做妇科B超检查排查其他疾病。医生还可能根据临床具体情况，进行其它相关实验室检查。以上所有检查均免费，这些将有助于医生更加全面细致地了解您的病情。这些检查都很安全，不会对您的健康和病情产生不良影响。

2. 若您通过以上筛查符合纳入标准，将按以下步骤进行研究：

（1）医生将根据随机数字表提供的随机信息，决定您接受“桂枝茯苓丸” 或“安慰剂组（安慰剂组使用的中医健脾开胃药物：炒麦芽）”。您分别有1/2的可能性被分入这2个不同组中的任何一个。您和您的治疗医生都无法事先知道和选择治疗方法。药物治疗为每次经前7天用药至月经第3日，连续给药3个月经周期。治疗结束后连续观察随访3个月经周期。研究总周期为6个月经周期，在筛选期、入组后每次月经结束后3天内各评价一次。

（2）治疗和随访期间，您需要详细如实记录您的用药情况。

（3）治疗结束后，观察医师会详细记录您的症状、体征。

（4）以上与本试验有关的治疗和检查均免费。

（5）本研究中使用的免煎剂药物由三峡大学附属第二人民医院药剂科提供。

3. 需要您配合的其他事项

您必须按医生和您约定的随访时间复诊。您的随访非常重要，因为医生将判断您接受的治疗是否真正起作用，并及时指导以预防您痛经的发作。同时，您还有责任向医生报告试验过程中您身体和精神方面的任何改变，无论您认为这种改变是否与这项研究有关。

研究期间请您按照医生推荐用药，并请您及时、客观地填写您的服药记录。

如您需要进行其它治疗，请事先与您的医生取得联系。

四、参加研究可能的受益

您将可能从本项研究中受益。此种受益包括您的病情有可能获得改善，如果您参加本研究，在研究期间，将得到相关的免费体格检查、免费 B 超检查和免费治疗。

五、参加研究可能的不良反应、风险和不适、不方便

本课题使用药物经大量临床验证副作用极少。如果在研究期间您出现任何不适。均应及时通知您的联系人，我们会及时作出判断并给与对应的医疗建议。

六、有关费用

医生将预防和治疗由于本研究可能带来的伤害。如果在临床试验中出现不良事件，医学专家委员会将会鉴定其是否与治疗或试验过程有关。申办者将参照我国《药物临床试验质量管理规范》的规定对与试验过程相关的损害提供治疗的费用及相应的经济补偿。

在治疗期间，对于您同时合并的其他疾病所需的治疗和检查，将不在免费的范围之内。

七、个人信息是保密的吗？

您的医疗记录（研究病历/CRF、化验单等）将完整地保存在您所就诊的医院。医生会将化验检查结果记录在您的病历上。研究者、伦理委员会和国家自然科学基金委员会相关部门将被允许查阅您的医疗记录。任何有关本项研究结果的公开报告将不会披露您的个人身份。我们将在法律允许的范围内，尽一切努力保护您个人医疗资料的隐私。

八、怎样获得更多的信息？

您可以在任何时间提出有关本项研究的任何问题，并得到相应的解答。

如果在研究过程中有任何重要的新信息，可能影响您继续参加研究的意愿时，您的医生将会及时通知您。

九、可以自愿选择参加研究和中途退出研究

是否参加研究完全取决于您的意愿。您可以拒绝参加此项研究，或在研究过程中的任何时间退出本研究，这都不会影响您和医生间的关系，都不会对您的医疗或其他方面利益的造成影响。

出于对您的最大利益考虑，医生或研究者可能会在研究过程中随时中止您继续参加本项研究。

如果您因为任何原因从研究中退出，您可能被询问有关您接受治疗的情况。如果医生认为需要，您也可能被要求进行实验室检查和体格检查。

十、现在该做什么？

是否参加本项研究由您自己（和您的家人）决定。

在您做出参加研究的决定前，请尽可能向你的医生询问有关问题。

感谢您阅读以上材料。如果您决定参加本项研究，请告诉您的医生，他/她会为您安排一切有关研究的事务。

请您保留这份资料。

知情同意书·同意签字页

临床研究项目名称：“桂枝茯苓丸”治疗热灼血瘀型原发性痛经的随机对照研究

课题承担单位：三峡大学第二人民医院

同意声明

我已经阅读了上述有关本研究的介绍，而且有机会就此项研究与医生讨论并提出问题。我提出的所有问题都得到了满意的答复。

我知道参加本研究可能产生的风险和受益。我知晓参加研究是自愿的，我确认已有充足时间对此进行考虑，而且明白：

- 我可以随时向医生咨询更多的信息。
- 我可以随时退出本研究，而不会受到歧视或报复，医疗待遇与权益不会受到影响。

我同样清楚，如果我中途退出研究，特别是由于药物治疗的原因使我退出研究时，我若将我的病情变化告诉医生，完成相应的体格检查和理化检查，这将对整个研究十分有利。

如果因病情变化我需要采取任何其他的药物治疗，我会在事先征求医生的意见，或在事后如实告诉医生。

我同意国家自然科学基金委员会、伦理委员会或申办者代表查阅我的研究资料。

我将获得一份经过签名并注明日期的知情同意书副本。

最后，我决定同意参加本项研究，并保证尽量遵从医嘱。

患者签名：__________________ __________年__________月__________日

联系电话：__________________

我确认已向患者解释了本试验的详细情况，包括其权力以及可能的受益和风险，并给其一份签署过的知情同意书副本。

医生签名：__________________ __________年__________月__________日

医生的工作电话：__________________
