# Supplementary material for: Efficacy of Guizhi Fuling Wan for primary dysmenorrhea: protocol for a randomized controlled trial
Source: Trials. 2021 Dec 18;22:933. doi: 10.1186/s13063-021-05834-0 (PMC8684114; doi:10.1186/s13063-021-05834-0)
Supplement: Supplementary file 4 — Additional file 4. Ethical Approval Document. [file 13063_2021_5834_MOESM4_ESM.pdf]

Ethical Approval Document

文件编号：2020001
版本日期：2020-5-26

宜昌市第二人民医院临床试验伦理委员会

科研立项项目伦理审查申请

项目名称	新制桂枝茯苓丸治疗热灼血瘀型原发性痛经的随机对照研究		
研究性质	☐ 纵向科研项目<br>☐ 国家级 ☐ 省级 ☐ 市级 ☐ 其他<br>批文批准号：<br>☑ 横向科研项目<br>☐ 自选科研项目		
研究类型	☑ 主持<br>☐ 协作：<br>主持单位名称：<br>主持单位伦理批件号及日期：2020001 2020.06.05		
研究目的	新制桂枝茯苓丸治疗热灼血瘀型原发性痛经的随机对照研究		
本中心研究科室/项目负责人、职称	李晨劼，医师	联系电话/邮箱	13085157861<br>179073543@qq.com
研究计划总例数/本中心承担例数	128/128	研究期限	2020年9月 12日至2022年12月31日

一、研究设计类型：

1. 是否为试验性研究：☑ 是 ☐ 否

2. 是否为观察性研究：☑ 是（☐ 回顾性分析 ☑ 前瞻性研究） ☐ 否

二、研究信息

1. 资金来源：☐ 企业 ☑ 政府 ☐ 学术团体 ☐ 本单位 ☐ 自筹

2. 研究是否需要使用人体生物标本：☑ 是（☑ 采集粪便 ☐ 利用以往保存的生物标本）
☐ 否

3. 受试者可能出现的不良反应（如有，请附说明）

请见附件1说明

4. 对不良事件的相应防治措施（如有，请附说明）

请见附件 1 说明

5. 受试者可能的风险及受益（如有，请附说明）

请见附件 2 说明

三、知情同意书信息

1. 知情同意书：☑ 有　☐ 无

2. 免除知情同意申请：

☐ 利用以往临床诊疗中获得的病历/生物标本的研究

☐ 研究病历/生物标本的二次利用

四、招募过程

1. 招募方式：☐ 广告　☐ 个人联系　☐ 数据库　☐ 中介　☑ 其他

2. 招募人群特征：☐ 健康者　☑ 患者　☐ 弱势群体　☐ 孕妇

3. 是否支付受试者交通费：☐ 是　☑ 否

五、提交的材料（请注明科研项目、知情同意书版本号和版本日期）

1. 研究方案(V1.0/2019 年 5 月 12 日)
2. 知情同意书(V1.0/2019 年 5 月 12 日)
3. 病例报告表(V1.0/2019 年 5 月 12 日)

项目负责人声明：

我证实本人与该研究项目不存在利益冲突，由我签名的这份研究申请报告的各项资料已由我检查审阅，并保证在临床研究实施过程中所有信息是真实的、准确的并符合科研项目的要求及国家相关法律法规的要求。

项目负责人<br>签名/日期	[illegible]　2020 年 6 月 5 日
伦理委员会<br>审查意见	经审查该项目，是/否违反伦理道德，同意/不同意其开展该项目。<br>伦理委员会签字 [illegible]（盖章）2020 年 6 月 5 日
