# Supplementary material for: Efficacy of Guizhi Fuling Wan for primary dysmenorrhea: protocol for a randomized controlled trial
Source: Trials. 2021 Dec 18;22:933. doi: 10.1186/s13063-021-05834-0 (PMC8684114; doi:10.1186/s13063-021-05834-0)
Supplement: Supplementary file 5 — Additional file 5. Funding Documentation. [file 13063_2021_5834_MOESM5_ESM.pdf]

Notice of Approval of the National Natural Science Foundation of China

Ms Xianyun Fu

According to the "Regulations of the National Natural Science Foundation of China" and expert review opinions, the National Natural Science Foundation of China (Here in after referred to as the Natural Science Foundation of China) has decided to approve your application for funding. Project approval number: 81973897, Project Title: Research on the Mechanism of Rhubarb-Peach Seed Medicine in Regulating Hypoxic Mitochondrial Homeostasis in Adenomyosis, Direct Cost: 560,000 yuan, project start and end date: January 2020 to December 2023, see annex for related project review comments and modifications..

Please visit the Science Foundation Network Information System (https://isisn.nsfc.gov.cn) as soon as possible to obtain the National Natural Science Foundation of China Funding Project Plan" (hereinafter referred to as the plan) and fill in as required. For items with revised opinions, please adjust the relevant content of the plan in time according to the revised opinions; if you have objections to the revised opinions, you must submit the proposal to the relevant science department before the deadline for submission of the electronic version of the plan.

The electronic version of the plan is uploaded through the Science Foundation Network Information System (https://isisn.nsfc.gov.cn) and submitted to the Natural Science Foundation of China for review after review by the supporting unit. Those who have not passed the review will be returned after modification, then submitted again; those who pass the review will print the paper plan (in duplicate, double-sided printing) and rely on the unit for review. After verification and affixed with the official seal of the unit, it shall be submitted to the Natural Science Foundation of China Project Material Receiving Working Group. The content of the Electronic version and paper version plan should be consistent. The deadlines for submission and filing of plans to the Chinese Natural Science Foundation are as follows:

1. The deadline for submitting the electronic version of the plan is 16:00 on September 11, 2019 (deemed as when the plan is officially submitted);

2. The deadline for submitting the electronic revised version of the plan is 16:00, September 18, 2019;

3. The deadline for submitting the paper plan is 16:00 on September 26, 2019.

Please submit the electronic version of the plan in time according to the above regulations, and submit the paper version of the plan. Those who do not give reasons and fail to submit the plan within the time limit shall be deemed to have automatically given up accepting the funding.

Attachment: Project Review Opinion and Modification Opinion Form

National Natural Science Foundation of China

August 16, 2019
